# Fine Tuning of Phosphorothioate Inclusion in 2′-O-Methyl Oligonucleotides Contributes to Specific Cell Targeting for Splice-Switching Modulation

**DOI:** 10.3389/fphys.2021.689179

**Published:** 2021-10-13

**Authors:** Yoshitsugu Aoki, Cristina S. J. Rocha, Taavi Lehto, Shouta Miyatake, Henrik Johansson, Yasumasa Hashimoto, Joel Z. Nordin, Imre Mager, Misako Aoki, McClorey Graham, Chaitra Sathyaprakash, Thomas C. Roberts, Matthew J. A. Wood, Mark A. Behlke, Samir El Andaloussi

**Affiliations:** ^1^Department of Molecular Therapy, National Institute of Neuroscience, National Center of Neurology and Psychiatry (NCNP), Tokyo, Japan; ^2^Department of Paediatrics, University of Oxford, Oxford, United Kingdom; ^3^Department of Laboratory Medicine, Clinical Research Center, Karolinska Institutet, Karolinska University Hospital, Huddinge, Sweden; ^4^Department of Oncology-Pathology, Clinical Research Center, Karolinska Institutet, Karolinska University Hospital, Huddinge, Sweden; ^5^Integrated DNA Technologies, Inc., Coralville, IA, United States

**Keywords:** skeletal muscle, Duchenne muscular dystrophy, splice-switching oligonucleotide (SSO), phosphorothioate, 2OMePS

## Abstract

Splice-switching antisense oligonucleotide- (SSO-) mediated correction of framedisrupting mutation-containing premessenger RNA (mRNA) transcripts using exon skipping is a highly promising treatment method for muscular diseases such as Duchenne muscular dystrophy (DMD). Phosphorothioate (PS) chemistry, a commonly used oligonucleotide modification, has been shown to increase the stability of and improve the pharmacokinetics of SSOs. However, the effect of PS inclusion in 2′-O-methyl SSOs (2OMe) on cellular uptake and splice switching is less well-understood. At present, we demonstrate that the modification of PS facilitates the uptake of 2OMe in H2k-*mdx* myoblasts. Furthermore, we found a dependency of SSO nuclear accumulation and high splice-switching activity on PS inclusion in 2OMe (2OMePS), as tested in various reporter cell lines carrying pLuc/705. Increased exon-inclusion activity was observed in muscle, neuronal, liver, and bone cell lineages *via* both the gymnotic uptake and lipofection of 2OMePS. Using the photoactivatable ribonucleoside-enhanced crosslinking and a subsequent proteomic approach, we identified several 2OMePS-binding proteins, which are likely to play a role in the trafficking of 2OMePS to the nucleus. Ablation of one of them, *Ncl* by small-interfering RNA (siRNA) enhanced 2OMePS uptake in C2C12 myoblasts and upregulated luciferase RNA splicing in the HeLa Luc/705 reporter cell line. Overall, we demonstrate that PS inclusion increases nuclear delivery and splice switching in muscle, neuronal, liver, and bone cell lineages and that the modulation of 2OMePS-binding partners may improve SSO delivery.

## Introduction

In the last few decades, numerous studies have demonstrated the potential of using splice-switching antisense oligonucleotides (SSOs). SSOs are typically 15–25 mers, short oligonucleotides, designed to target pre-messenger RNA (mRNA) and modulate the splicing patterns of target transcripts by blocking Watson–Crick RNA–RNA base-pairing or RNA–protein-binding interactions (Zamecnik and Stephenson, 1978; Dominski and Kole, 1993). This function gives SSOs a solid therapeutic potential, particularly, for exon skipping in Duchenne muscular dystrophy (DMD). SSOs using 2′-O-methyl RNA (2OMe) chemistry designed to restore dystrophin were the first oligonucleotides tested in DMD (Cirak et al., 2011). However, they failed at trials due to only a marginal clinical benefit for patients, including low efficiency of dystrophin restoration to the muscle. However, 2OMe SSOs have a tremendous promise as a treatment, and further optimisation and understanding of its delivery mechanism and action are crucial for use in related diseases.

A major limitation of the unmodified SSO using 2OMe in a therapeutic context is its instability in biological fluids due to susceptibility to nuclease digestion. The introduction of phosphorothioate (PS) linkages, replacing a phosphodiester backbone, was a commonly used oligonucleotide modification, which dramatically increased SSO stability and improved their ability to interact with serum proteins, including albumin, increasing their half-life and bioavailability (Dias and Stein, 2002). Subsequent SSO designs typically display 2′ modifications in the ribose ring, such as a naturally occurring methyl group. Most of these modifications further increase oligonucleotide stability and significantly enhance their binding affinity toward complementary RNAs. For *in vitro* and *in vivo* splice-switching applications, 2OMe has been extensively used with full PS modifications (Heemskerk et al., 2010; Ezzat et al., 2012; Flanigan et al., 2014). It has been shown that three-terminal PS entities on each side of SSOs are sufficient to confer exonuclease resistance (Mathy et al., 2007). However, the optimal modifications of PS inclusion in 2OMe to improve the splicing efficiency of SSO are not well-understood.

The activity of 2OMePS depends on numerous factors, including RNA secondary and tertiary structures, protein binding with target RNAs, the subcellular localization of 2OMePS, the interaction of 2OMePS with endogenous intracellular proteins and cellular machinery, and cellular uptake. An understanding of the interaction of 2OMePS with proteins may facilitate the development of more efficacious SSOs. PS-modified 2OMe is known to bind more proteins and to have a higher affinity than 2OMe with a phosphodiester backbone (Dias and Stein, 2002). Differences in protein binding can result in different pharmacological profiles and alter the subcellular distribution of SSOs (Weidner et al., 1995; Baltz et al., 2012). Although some proteins, such as nucleolin (NCL), albumin, and chaperonin T-complex 1 (TCP1) that interact with 2OMePS, have been previously identified (Liang et al., 2014), the effects of these proteins on 2OMePS activity and subcellular localization remain elusive.

Here, we aimed to fine-tune PS inclusion in 2OMe to improve SSO splicing efficiency in several cell types. Muscle, neuronal, liver, and bone cell lines were investigated to understand whether different target cell types would pose respective challenges in the case of the same SSO. We further investigated the interaction of PS-modified 2OMe with the other cellular proteins in C2C12 myoblasts.

## Materials and Methods

### Splice-Switching Antisense Oligonucleotides

All SSOs targeting the donor splice site of exon 23 of the mouse *Dmd* pre-mRNA with PS- and end2OMePS modifications (5′-GGCCAAACCUCGGCUUACCU-3′) were synthesized by Integrated DNA Technologies (IDT, Coralville, IA, USA). Cy3- or FITC-conjugated 2OMePS and 2OMe-endPS were also synthesized by IDT. The nomenclature, size, and sequence of all SSOs used in this study are summarized in Table 1.

**Table 1:** Nomenclature, size, and sequence of oligonucleotides.

**Name**	**Size (mers)**	**Sequence (5′-3′)**
PS25	25 (+7,−18)	G^*^G^*^C^*^C^*^A^*^A^*^A^*^C^*^C^*^U^*^C^*^G^*^G^*^C^*^U^*^U^*^A^*^C^*^C^*^U^*^G^*^A^*^ A^*^A^*^U
PS20	20 (+2,−18)	G^*^G^*^C^*^C^*^A^*^A^*^A^*^C^*^C^*^U^*^C^*^G^*^G^*^C^*^U^*^U^*^A^*^C^*^C^*^U
endPS20	20 (+2,-18)	G^*^G^*^C^*^CAAACCUCGGCUUA^*^C^*^C^*^U
PS18 (705)	18	C^*^C^*^U^*^C^*^U^*^U^*^A^*^C^*^C^*^U^*^C^*^A^*^G^*^U^*^U^*^A^*^C^*^A
endPS18 (705)	18	C^*^C^*^U^*^CUUACCUCAGUU^*^A^*^C^*^A
PS18-alt (705)	18	C^*^CU^*^CU^*^UA^*^CC^*^UC^*^AG^*^UU^*^AC^*^A
PS18-11 (705)	18	C^*^C^*^U^*^C^*^U^*^U^*^ACCUCAG^*^U^*^U^*^A^*^C^*^A
PS18-9 (705)	18	C^*^C^*^U^*^C^*^U^*^UACCUCAGU^*^U^*^A^*^C^*^A
PS16 (705)	16	C^*^U^*^C^*^U^*^U^*^A^*^C^*^C^*^U^*^C^*^A^*^G^*^U^*^U^*^A^*^C
endPS16 (705)	16	C^*^U^*^C^*^UUACCUCAGU^*^U^*^A^*^C
PS-4-thiodT	20 (+2,−18)	Biotinylated 5′-G^*^G^*^C^*^C^*^A^*^A^*^A^*^C^*^C^*^U^*^C^*^G^*^G^*^C^*^U^*^U^*^A^*^C^*^C^*^U−3′−4-thiodT
2OMe endPS-4-thiodT	20 (+2,−18)	Biotinylated 5′-G^*^G^*^C^*^CAAACCUCGGCUUA^*^C^*^C^*^U-3′−4-thiodT

* *Phosphorothioate linkage*.

### Cell Line Culture Conditions

C2C12 and HeLa cells were grown in Dulbecco's Modified Eagle Medium (DMEM), high glucose, GlutaMAX media (Life Technologies, Carlsbad, CA, USA) with 10% fetal bovine serum (FBS) (Life Technologies, Carlsbad, CA, USA) and 1% penicillin/streptomycin (Life Technologies, Carlsbad, CA, USA). Murine H2k-*mdx* myoblasts were cultured in gelatin- (0.01%) coated flasks at 33°C, under 10% CO_2_ in DMEM supplemented with 20% heat-inactivated FBS (FBS Gold, PAA Laboratories, Pasching, Austria), 2% chicken embryo extract (Seralab, Sussex, UK), 1% penicillin/streptomycin-neomycin antibiotic mixture (Life Technologies, Carlsbad, CA, USA), and 3 pg/ml γ-interferon (PeproTech, Rehovot, Israel). For differentiation, the cells (1 × 10^5^) were seeded into the wells of a 24-well plate, and the medium was changed after 24 h into a differentiation medium consisting of DMEM containing 2% horse serum (Life Technologies, Carlsbad, CA, USA) and was differentiated for 3–4 days before experimentation. For the SSO treatment, the cells were treated in the serum-free Opti-MEM® medium for 4 h, the medium was then changed for a differentiation medium, and incubation continued for 20 h. Reporter cell lines HeLa Luc/705, HuH7_705, C2C12_705, Neuro-2a_705, and U-2 OS_705 were cultivated and maintained in DMEM, high glucose plus 10% FBS with 200 (U-2 OS_705) or 400 (Huh7_705, C2C12_705, and Neuro-2a_705) μg/ml G418 (Life Technologies, Carlsbad, CA, USA) at 37°C, 5% CO_2_ in 95% humidity (Rocha et al., 2016).

### SSO or Small-Interfering RNA Delivery

Transfection with different concentrations of SSOs was performed with PepFect14 (Psyclo Peptide, Inc., Shanghai, China) (Ezzat et al., 2011) or Lipofectamine® 2000 (LF2000) (Life Technologies, Invitrogen, CA, USA) or OptiMEM (gymnotic delivery), which was formulated in OptiMEM for 15 min at room temperature according to the protocols of the manufacturer, and was then added to the cells cultured in DMEM + 10% FBS. The complexes were left in the culture for 24 h, after which the cells were harvested for RNA isolation or luciferase measurement. Transfection with 5 nM of Silencer Select siRNA® (Life Technologies, Carlsbad, CA, USA), including NCL s70420 and TCP1 s224715, was performed with LF2000, formulated in OptiMEM 15 min at room temperature, according to the protocols of the manufacturer, and was then added to the cells cultured in DMEM + 10% FBS. The complexes were left in the culture for 48 h, after which the cells were harvested for RNA isolation.

### RNA Expression Analysis

Total RNA was isolated using Tri-Reagent® (Sigma-Aldrich, St. Louis, MO, USA) according to the protocol of the manufacturer. Total RNA quantity and quality were analyzed by NanoDrop 2000 (Thermo Scientific, Waltham, MA, USA). For complementary DNA (cDNA) synthesis, 500 ng of total RNA was used with the High-Capacity cDNA Reverse Transcription kit (Applied Biosystems, Warrington, UK) according to the protocol of the manufacturer. Reverse transcription PCR (RT-PCR) was performed with 12.5 ng of cDNA in each reaction (the total volume per reaction was 25 μl) using the HotStarTaq Plus DNA polymerase kit (QIAGEN, Hilden, Germany) following the protocol of the manufacturer. Primers targeting luciferase mRNA had the following sequences. Forward primer 5′-TTGATATGTGGATTTCGAGTCGTC-3′; reverse primer 5′-TGTCAATCAGAGTGCTTTTGGCG-3′ (CyberGene, Solna, Sweden); and the PCR program employed was 5 s 95°C; 30 s 95°C, 30 s 55°C, 30 s 72°C for 29 cycles; and 10 min at 72°C for the final extension. The PCR products were analyzed on a 2% agarose gel in 0.5 × Tris-Borate-EDTA buffer and visualized by SYBRsafe (Invitrogen, Carlsbad, CA, USA) staining. Gel images were captured on a Fluor-S gel documentation system (Bio-Rad, Hercules, CA, USA) with the Quantity One software (Bio-Rad, Hercules, CA, USA). Quantitative PCR (qPCR) analysis was performed on cDNA from C2C12 and H2k-*mdx* cells using the cDNA template (25 ng) and amplified by the TaqMan Gene Expression Master Mix (Applied Biosystems, Waltham, MA, USA) on a StepOne Plus Thermocycler (Applied Biosystems, Waltham, MA, USA). TaqMan probes targeting Ncl1 and Tcp1 (Life Technologies, Carlsbad, CA, USA) were used, and murine glyceraldehyde 3-phosphate dehydrogenase (Gapdh) probes were used as an internal control for cDNA levels.

### Luciferase Assay

To measure luciferase activity, the medium was removed, the wells were washed two times with 1 × phosphate-buffered saline (PBS), and the cells were lysed in 150 or 25 ml of 1 × PBS with 0.1% Triton-X 100 per well for 24- or 96-well plates, respectively. The cells were incubated for 20 min at 4°C, followed by a frost/defrost cycle at −80°C. After this, the lysates were kept on ice until use. About 20 μl of the lysates were mixed by an injector with 100 ml of the luciferase assay reagent (1 mM EDTA pH 8.0, 20 mM Tricine pH 7.8, 1 mM MgCO_3_ pH 7.8, 5 mM MgSO_4_, 25 mM 1,4-dithiothreitol, 1 mM adenosine 5′-triphosphate disodium salt hydrate, 25 mM coenzyme A, and 1 mM d-luciferin). The relative light units of luciferase were determined using the GloMax® 96 Microplate Luminometer (Promega, Madison, Wisconsin, USA) with an integration time of 10 s. The values were normalized by the total protein quantity that was determined using the DC Protein Assay (Bio-Rad, Hercules, CA, USA).

### Immunostaining and Fluorescence Microscopy

For immunofluorescence, the cells were treated with Cy3- or FITC-conjugated 2OMePS oligonucleotides for 4 h, washed three times with PBS containing Ca^2+^ and Mg^2+^ solution, and fixed with methanol at −20°C for 10 min. Then, the cells were washed and stored in PBS at 4°C for future immunofluorescence analysis. For colocalisation, the cells were treated with 0.1% Triton-X100 (Sigma-Aldrich, München, Germany) in PBS for 10′, washed three times with PBS plus, and then blocked with PBS containing 1% bovine serum albumin (BSA, Sigma-Aldrich, St. Louis, MO, USA) for 1 h. After this, the cells were incubated with rat anti-mouse NCL antibody (1:200 dilution, Bio-Rad, Hercules, CA, USA), washed three times with PBS plus, and treated with 1:500 Alexa Fluor 488 goat anti-rat antibody (Life Technologies, Carlsbad, CA, USA) for 1 h. 4′,6-diamidino-2-phenylindole (DAPI) (1:5000 dilution, Sigma-Aldrich, St. Louis, MO, USA) staining, which was then performed for 2′, after which the cells were washed and mounted with the fluorescent mounting medium S3023 (Dako, Tokyo, Japan) onto glass slides. The visualization was carried out on a Leica fluorescent microscope, and the pictures were taken by an Axiovision fluorescent camera and the Axiovision software (Zeiss, Oberkochen, Germany).

### UV Crosslinking, Immunoprecipitation, and Nano-High Performance Liquid Chromatography

We applied only the photoactivatable ribonucleoside-enhanced crosslinking and immunoprecipitation (PAR-CLIP) protocol that uses photoactivatable 4-Thio-Dthymidine (4ThioT). C2C12 myoblasts (1 × 10^7^ cells) were transfected with 5′-biotin-2OMePS-4ThioT-3′ or 5′-biotin-2OMe-endPS-4ThioT-3′ (100 nM as the final concentration) under gymnotic uptake settings for 30 min and then rinsed to remove excess oligo. UV crosslinking was performed using Stratalinker® 2400 Bulbs, 365 nm (Stratagene, San Diego, CA, USA). The cells were harvested using a silicon scraper and lysed in the plate with lysis buffer 1 (LB1: 10 mM of 1 M Hepes-KOH, pH 7.5, 100 mM of 5 M NaCl, 1 mM of 0.5 M EDTA, 0.5 mM of 0.5 M EGTA, 0.1% of sodium-deoxycholate, and 0.5% of sodium lauroyl sarcosinate) using a protease inhibitor cocktail tablet from Complete. The cell pellet was resuspended in 1 ml LB1 and sonicated [six cycles of 30 s with 1-min intervals at power 7.0 (~40 watts)]. Streptavidin beads (Dynabeads MyOne Streptavidin C1, Thermo Fisher, Waltham, MA, USA) were mixed with the supernatant at 4°C O/N. The beads were washed, transferred to a clean 1.5 ml tube, and boiled in lithium dodecyl sulfate loading buffer, and the supernatant was collected in a new 1.5 ml, following the instructions of the manufacturer.

The supernatant was fractionated and analyzed on a nano-high performance liquid chromatography system (Proxeon, Seattle, WA, USA) coupled directly to a Linear Trap Quadrupole Orbitrap Velos (Thermo Fisher Scientific, Waltham, MA, USA). The identification and quantification of proteins were processed by standard protocols. A detailed description of the protein identification and quantification can be found in the Supplemental Material.

### Biolayer Interferometry and Dissociation Constant (KD) Calculation

The BioLayer Interferometry technology (BLItz) uses optical biosensors to measure multiple interactions in parallel without detection agents. Recombinant NCL (RPC242Mu01, Cloud-Clone Corp, Katy, TX, USA), a fusion protein of Tyr353–Ser568 of NCL and an N-terminal His-Tag (NCL-His), was used for this BLItz label-free protein assay. Before its use, biosensors were soaked in the BLItz assay buffer (20 mM Tris-HCl, pH 8.0, 150 mM KCl, 0.02% Tween 20, 2 mM DTT, and 1 mg/ml BSA) for at least 10 min. Biolayer interferometry assays consisted of five steps, all performed in the BLItz assay buffer: initial baseline (30 s), loading (120 s), baseline (120 s), association (120 s), and dissociation (120 s). NCL-His was immobilized on the Ni-NTA biosensor. For the loading step, biotinylated 2OMePS and 2′OMe concentrations (0, 1.56, 3.13, 6.25, 12.5, 25, and 50 mM) were adjusted to yield a signal intensity in the range of 0–0.1 nm, thereby ensuring that the sensors were not saturated. Control values, measured using empty (no NCL-His loaded) sensors, were subtracted from experimental values before data processing. Initial experiments indicated that empty sensors and sensors loaded with control biotin-labeled DNA yielded similar values in binding experiments with MBP-R1c. The equilibrium KD rate constant was calculated.

### Data Analysis

Data were expressed as mean ± SEM. Values were tested for normality by the D'Agostino-Pearson normality test (omnibus K2). Statistical significance was determined by one-way ANOVA followed by a comparison of each treatment group with the control group by Fisher's least significance difference (LSD) test (GraphPad Prism 6 Software, GraphPad Software, Inc., San Diego, CA, USA). In all cases, *p* < 0.05 was defined as significant. The detection of differentially expressed proteins is based on a 5% false discovery rate (5% FDR).

## Results

### Splice Switching Is Dependent on PS Inclusion in 2OMe Upon Lipofection in Muscle, Neuronal, Liver, and Bone Reporter Cell Lineages

**Figure 1:**
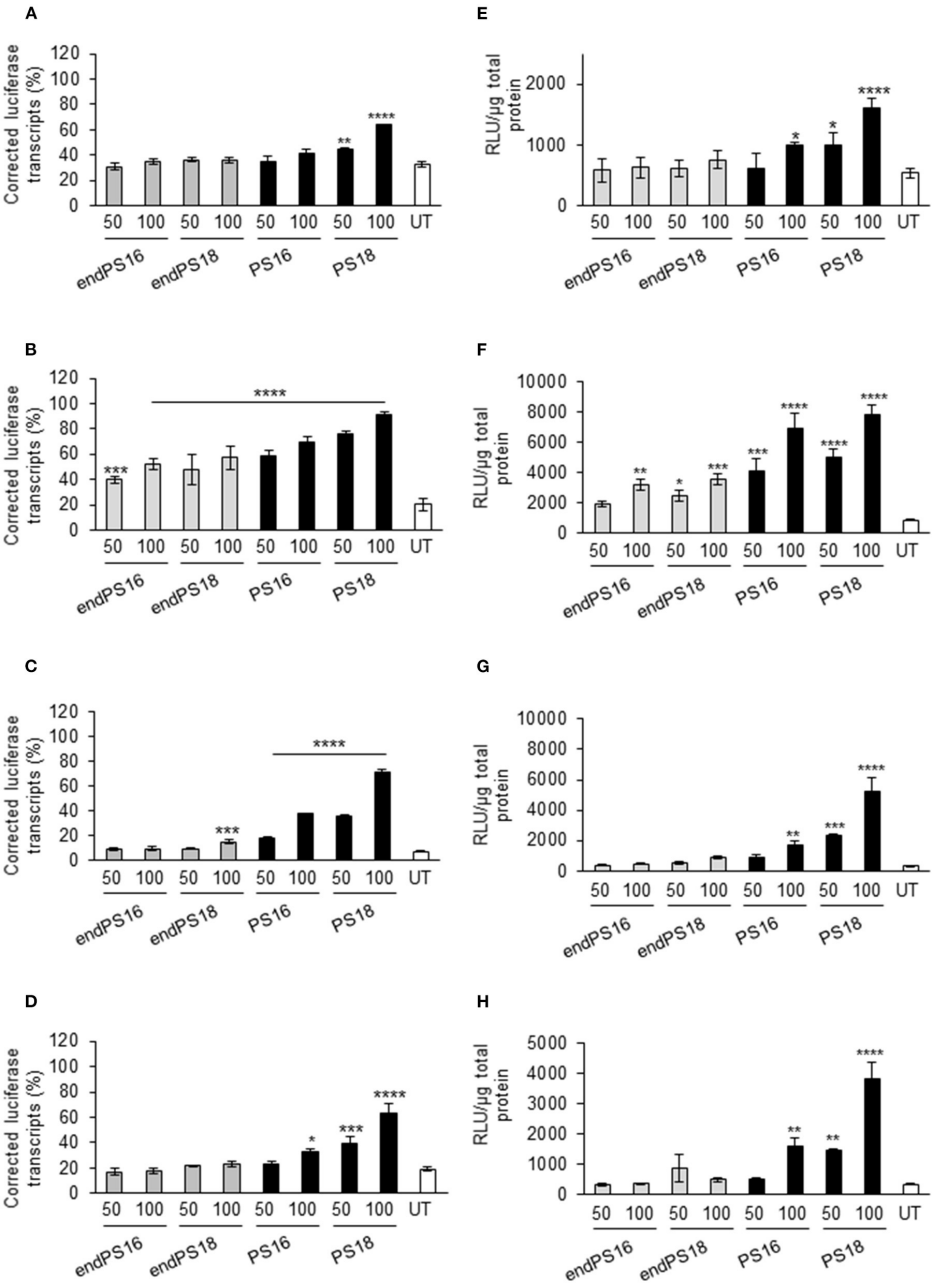
Phosphorothioate (PS) modification modulates the splice-switching activity in different cellular models. Reverse transcription PCR of aberrant and correct luciferase mRNA and 18S RNA in all reporter cell lines when the concentrations of 50 and 100 nM of 18 or 16 mers were modified with full PS vs. endPS 2OMe were delivered by PepFect14 nanoparticles and the effect measured after 24 h. Graphs represent the percentage of correct transcript compared with total pLuc705 mRNA transcribed after 24 h post-treatment in C2C12 _705 **(A)**, U-2 OS_705 **(B)**, Neuro-2a_705 **(C)**, and HuH7_705 **(D)** reporter cell lines when transfected with the concentrations of 50 and 100 nM of endPS or PS 2OMe. Graphs represent the relative luminescence units (RLU) normalized by micrograms of total protein from C2C12 _705 **(E)**, U-2 OS_705 **(F)**, Neuro-2a_705 **(G)**, and HuH7_705 **(H)** reporter cell lines when transfected at the concentrations of 50 and 100 nM of endPS or PS 2OMe. Data represent the mean ± SEM obtained from the three independent experiments. UT: Untreated. *p* < 0.05 was defined as statistically significant. *p < 0.05; **p ≤ 0.01, ***p ≤ 0.001; ****p ≤ 0.0001.

To date, exon-skipping SSOs with PS modifications have been primarily studied in muscle cells due to their clinical relevance in neuromuscular diseases. However, their application to other cell types has been overlooked. Thus, we briefly investigated whether the PS-dependent activity remains consistent in a greater variety of relevant reporter cell lines to enhance tissue-specific uptake. This may provide a better understanding of whether SSOs require specific chemical modifications for tissue targeting, in addition to enhancing stability and half-life. To this end, we used the four pLuc/705 splice-switching reporter cell lines derived from muscle, neuronal, liver, and bone cell lineages (C2C12, Neuro-2a, HuH7, and U-2 OS, respectively; Rocha et al., 2016), carrying the pLuc/705 splice-switching reporter (Supplementary Figure 1). In this reporter system, the presence of a defective intronic 5′ splice site that activates a cryptic 3′ splice site results in aberrant splicing of luciferase pre-mRNA and the translation of a non-functional luciferase reporter. An SSO targeted to the 5′ splice site masks the mutation, allowing the splicing machinery to be redirected and generating the corrected mRNA to restore luciferase activity. To investigate the optimal modifications of PS inclusion in 2OMe to improve the splicing efficiency of SSO, 16- and 18-mer 2OMe with PS or endPS modification at a final concentration of 50 or 100 nM were transfected into the reporter cell lines by PepFect14 following our previously published protocols (Ezzat et al., 2011; Rocha et al., 2016). We found that the percentage of the corrected luciferase transcript compared with total pLuc705 mRNA transcript (Figures 1A–D) and the relative luminescence units (RLU) normalized by micrograms of total protein (Figures 1E–H) were quantitatively increased relatively in a dose-dependent manner in C2C12_705, Neuro-2a_705, HuH7_705, and U-2 OS_705 cell lines. Notably, exon-skipping was far more efficient in the U-2 OS_705 line across all the examined PS-SSOs compared with C2C12_705, suggesting that myoblasts might contain an inherent hurdle for SSOs. We also found that 2OMePS with 18 mers rather than with 16 mers resulted in increased splice switching. Furthermore, splice switching in the HeLa_705 cell line was highly dependent on the PS substitution degree in 2OMe (Supplementary Figure 2A), and the effects were mediated in a dose-dependent manner (Supplementary Figure 2B).

Overall, 2OMePS oligonucleotides are taken up and efficacious at inducing splice switching in various reporter lines, with a moderate variability between lines. This suggests that subtle differences in chemical modifications in SSOs may be crucial in modulating the transcript correction though the experiments have given now cannot predict how more complex cellular interactions may affect them *in vivo*.

### PS Modification Facilitates the Uptake of 2OMe in Skeletal Muscle Cells

As a next step, to further assess the impact of PS modification on the uptake and exon-skipping activity of 2OMe in dystrophic muscle cells, we chose to use murine H2k-*mdx* myoblast, a widely studied myoblast model of DMD. We used a 20-mer sequence (Ex23D +2–18), previously optimized in mice, as a parent sequence for our modified SSO designs (Mann et al., 2002) before applying our findings to mice *in vivo* as a future study.

It has been indicated that only three-terminal PS modifications are necessary to confer the full exonuclease stability of 2OMe (Lennox et al., 2013). 2OMe SSO is also known to distribute differently within the cells depending on the applied delivery method (Dias and Stein, 2002). We synthesized both Cy3-labeled 20-mer 2OMePS containing a full PS backbone (Cy3-PS20) and Cy3-labeled low-PS-versions (Cy3-endPS20). We transfected H2k-*mdx* myoblasts with Cy3-endPS20 or Cy3-PS20 using either LF2000, which was previously optimized for the cells, or on gymnosis (Ezzat et al., 2011). Their uptake and cellular localization were observed by fluorescent microscopy.

**Figure 2:**
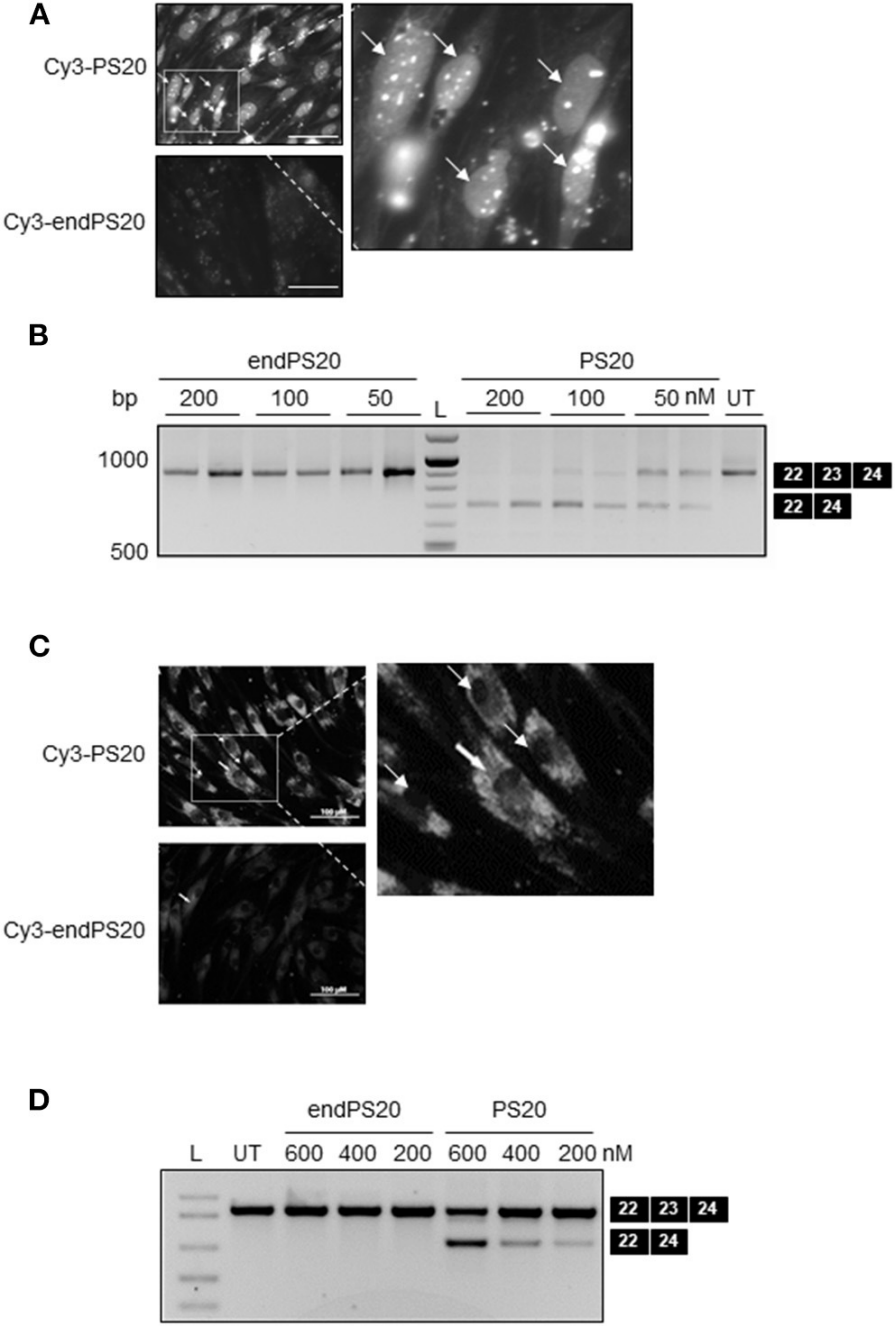
PS-dependent cellular uptake and nuclear accumulation of 2OMe modified splice-switching antisense oligonucleotides (SSOs) in H2k-*mdx* myoblasts. Cy3-conjugated 2OMePS or 2OMe-endPS SSO with 20 mers were delivered either through gymnotic uptake or by LF2000. **(A)** Fluorescence microscopy images depicting the cellular uptake of 2OMePS or 2OMe-endPS SSO (200 μM final concentration) complexed with Lipofectamine 2000 in H2k-*mdx* myoblasts after 4 h post-transfection. Bar = 15 μm. **(B)** RT-PCR depicting splice-switching activity of 2OMePS or 2OMe-endPS SSO in H2k-*mdx* myoblasts targeting exon 23 skipping of dystrophin with LF2000. UT: untreated. **(C)** Fluorescent microscopy images depicting the gymnotic cellular uptake of 2OMePS or 2OMe-endPS SSO (200 μM final concentration) in H2k-*mdx* myoblasts 4 h post-transfection. Bar = 15 μm. **(D)** RT-PCR depicting the splice-switching activity of 2OMePS or 2OMe-endPS SSO in H2k-*mdx* myoblasts targeting exon 23 skipping of dystrophin upon gymnotic uptake. L, DNA ladder; endPS, 2OMe-endPS. PS20 denotes 2OMePS with 20 mers.

Cy3-PS20 was rapidly taken up by H2k-mdx myoblasts, 4h post-transfection (Figure 2A). Fluorescence microscopy revealed primary accumulation at the nucleus, in a diffuse form or a distinct nuclear dot-like pattern, known as PSbodies. However, endPS20 showed negligible uptake. We then transduced H2k-*mdx* myoblasts with endPS20 or PS20 at a final concentration of 50, 100, or 200 nM, and exon 23 skipping efficiency was evaluated by RT-PCR after 48- h incubation. We found that endPS20 remained completely inactive, but PS20 induced 40–100% exon 23 skipping dose-dependently in H2k-*mdx* myoblasts (Figure 2B).

Next, we sought to carry out similar uptake and exon-skipping assessments in the context of gymnotic cellular uptake. In contrast to lipofection, 4 h after gymnotic uptake Cy3-PS20 exhibited a predominant cytosolic distribution in H2k-mdx myoblasts, examined by fluorescence microscopy while endPS20 showed negligible uptake (Figure 2C). We then transduced H2k-*mdx* myoblasts with endPS20 or PS20 at a final concentration of 200, 400, or 600 nM, and exon 23 skipping efficiency was evaluated by RT-PCR after 48 h incubation. We found that endPS20 remained completely inactive, but PS20 induced 20–50% exon 23 skipping dose-dependently in H2k-*mdx* myoblasts (Figure 2D).

Collectively, these data support the hypothesis that the full PS modification of 2OMe is a key in facilitating the uptake and activity on both lipofection and gymnotic transfection in H2k-*mdx* myoblasts. Furthermore, the lipofection and gymnotic transfection of 2OMe might lead to a predominant nuclear and cytosolic distribution in the cells, respectively.

### Determination of 2OMePS-Binding Proteins by Interactome Capture Studies in Living C2C12 Myoblasts

To provide better insights into the potential cellular uptake mechanisms of 2OMePS, we sought to identify the endogenous proteins that are in association with 2OMePS by using photoactivatable-ribonucleoside-enhanced crosslinking and immunoprecipitation (PAR-CLIP) to define the SSO interactome of uptake-permissive cells in a physiological environment (Castello et al., 2012, 2013). PAR-CLIP was originally developed to identify RNA-binding proteins and microRNA target sites. We have adapted it to identify the proteins interacting with SSOs, by performing UV crosslinking of 2OMePS-binding proteins to a 3′-4ThioT-coupled biotinylated 2OMePS (5′-biotin-2OMePS-4ThioT-3′) in living C2C12 myoblasts (as opposed to lysed cell-incubating 2OMePS) and captured the covalently bound proteins with magnetic Streptavidin-Dynabeads (Castello et al., 2013). After stringent washes, interacting proteins were detected by nano-high performance liquid chromatography. We identified and further validated 574 2OMePS-binding proteins (Supplementary Table 1) while we identified 32 proteins in the control group. In parallel, we confirmed that the biotinylated 2OMePS with photocrosslinking agents was able to induce efficient exon-23 skipping in C2C12 myoblasts with the same doses as nonbiotinylated 2OMePS without photocrosslinking agents (Supplementary Figure 3).

**Figure 3:**
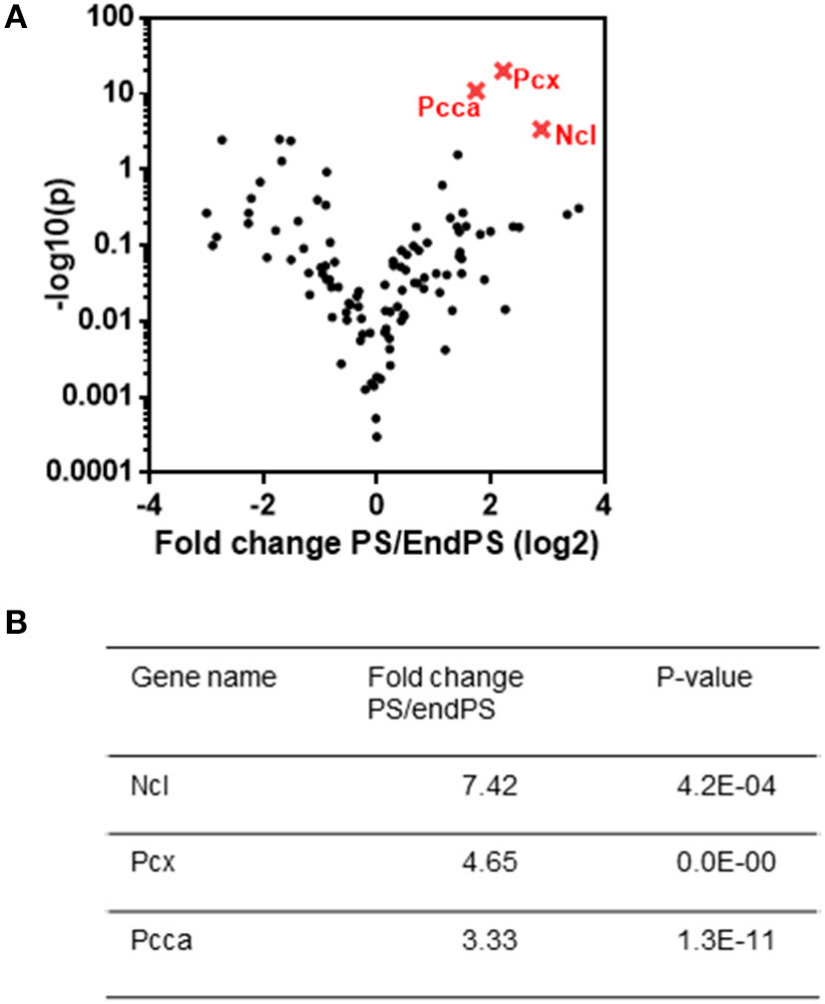
Crosslinking and proteomic analysis of proteins responsible for PS binding. **(A)** Comparison of PS and endPS UV+ sample interactome by a volcano plot analysis. **(B)** List of three statistically differentially expressed proteins (nucleolin (NCL), pyruvate carboxylase (PCX), and propionyl-CoA carboxylase subunit alpha (PCCA), which are found to be explicitly enriched in PS interactome as compared to endPS interactome. The most significantly enriched partners of the 2OMePS analog were identified by a volcano plot.

Following the differential expression analysis between PS20 and endPS20 UV+ samples, the most significantly enriched interacting partners of the 2OMePS analog were identified by a volcano plot (Figure 3A). We found that Ncl (fold change 7.42, *p* ≤ 0.0005), an abundant nucleolar phosphoprotein, was strongly associated with PS20 (Figure 3B). Mitochondrial enzymes, including pyruvate carboxylase (PCX) (fold change: 4.65, *p* ≤ 0.0001) involved in gluconeogenesis, adiposity, and insulin resistance in type 2 diabetes (Schär et al., 2010), and PCCA (fold change: 3.33, *p* ≤ 0.0001) (Figure 3B), were also the strong candidates for a PS20 interaction. Due to the supporting data from previous studies and the enrichment of Ncl being the highest among the three identified proteins, we focused our further investigations on unraveling the link between Ncl and PS20.

### Ncl Interacts With 2OMePS and May Regulate Its Antisense Activity

Ncl and Tcp1 have previously been shown to interact with 2OMePS and to colocalise in induced nuclear bodies in mammalian cells (Liang et al., 2014). Therefore, to study the role of Ncl in the uptake and biological activity of PS20, we treated the C2C12 myoblasts with small-interfering RNAs (siRNAs) targeting *Ncl* or *Tcp1* transcripts to deplete the expression of these proteins. It was reported that the estimated half-life of the surface Ncl was about 45 min and that of nuclear Ncl is more than 8 h (Hovanessian et al., 2010). Therefore, the treatment with siRNAs was performed 24 h prior to PS20 to confirm the effects resulting from Ncl depletion. Subsequently, C2C12 myoblasts were transfected with FITC-labeled PS20 (FITC-PS20) for 4 h, and the cellular uptake of FITC-PS20 was evaluated by fluorescent microscopy. The knockdown level of *Ncl* by qPCR was 67%, accompanied by the reduced expression of Ncl protein in *Ncl* siRNA-treated C2C12 myoblasts compared to the scramble treated ones (Supplementary Figures 4A,B).

**Figure 4:**
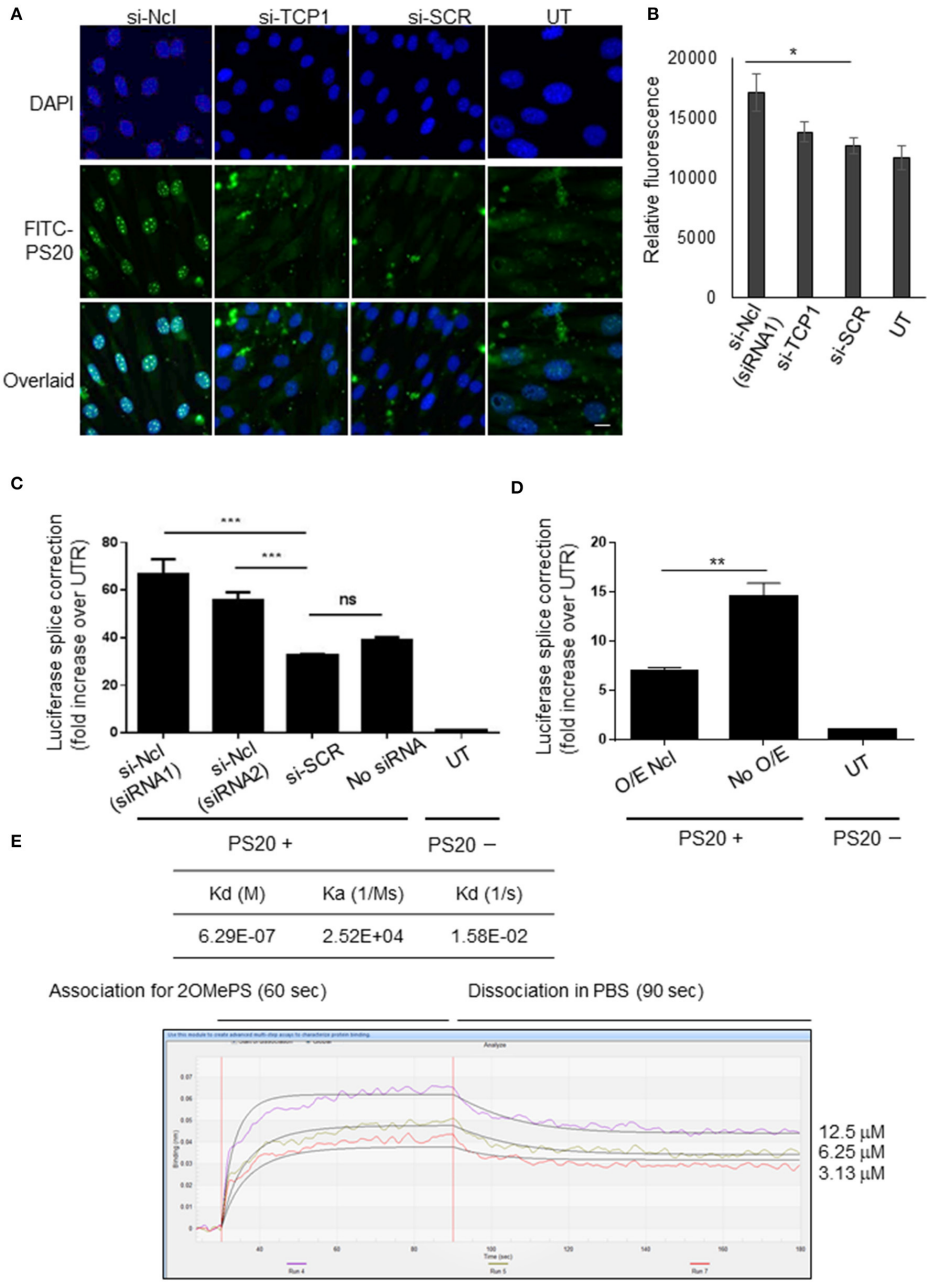
Nucleolin negatively regulates the cellular uptake and splicing efficiency of PS-modified 2OMe SSOs. **(A)** Nucleolin knockdown by siRNA enhances the cellular uptake of FITC-conjugated 2OMe splice-switching oligonucleotides (SSOs) in C2C12 myoblasts. Cells are incubated with FITC-conjugated 2OMePS for 4 h followed by an analysis using a fluorescent microscope. Bar = 15 μm. UT, untreated cells. **(B)** Relative fluorescent units of FITC-conjugated 2OMePS 4 h post-treatment in C2C12 myoblasts pretreated with siRNA against *Ncl* or *Tcp1*. **(C)** Effect of knockdown of *Ncl* or *Tcp1* on splicing efficiency in HeLa_705 with 2OMe SSO. **(D)** Ncl protein overexpression and impact on SSO activity upon lipofection. HeLa_705 cells are transfected with Ncl-encoding plasmid DNA (pDNA) for 24 h. Then, these and control cells are further treated with 2'-OMePS for 24 h, after which splice-switching activity was measured. O/E Ncl: the activity of lipofected SSOs in Ncl overexpressing cells. *P* < 0.05 was defined as statistically significant. *p < 0.05; **p ≤ 0.01, ***p ≤ 0.001. **(E)** BLItz label-free protein assays. The recombinant Ncl (RPC242Mu01, Cloud-Clone Corp, Katy, TX, USA), a recombinant protein of amino acids Tyr353–Ser568 of Ncl and an N-terminal His-Tag (NCL-His), was used for these assays. KD, the equilibrium dissociation constant between an antibody and its antigen.

Interestingly, FITC-PS20 uptake was significantly increased in the Ncl-depleted myoblasts while *Tcp1* silencing did not affect the uptake as measured by relative fluorescence intensity (Figures 4A,B). Splicing efficiency in a HeLa_705 luciferase reporter cell line transfected with siRNAs targeting Ncl 24 h prior to PS20 treatment was also correspondingly increased compared to control scramble-treated cells (Figure 4C). To further confirm the impact of Ncl on PS20 activity, HeLa_705 cells were transfected with a DNA plasmid encoding for Ncl overexpression for 24 h and subsequently treated with PS20 *via* lipofection (LF2000) for 24 h, after which the splice-switching activity was measured. Corroborating the siRNA data, we found that increased levels of Ncl have a negative impact on the splice-switching activity of PS20 in HeLa_705 cells (Figure 4D). We further confirmed the binding of a recombinant protein of Tyr353-Ser568 of Ncl with an NCL-His, which contains the RNA recognition motifs (Weidner et al., 1995), to PS20 using the BLItz (Figure 4E). We measured the equilibrium dissociation constant (Kd) between Ncl and PS20 and confirmed that the Kd value between Ncl and PS20 was 6.29E-07 M (Figure 4E). The data suggest an interaction between Ncl and PS20, which is compatible with the previous report that the binding of 2OMePS to Ncl occurs in a non-sequence-specific manner (Weidner et al., 1995). Together, our data indicate that Ncl interacts with 2OMePS and could negatively regulate the cellular uptake of 2OMePS and the splicing efficiency in mouse C2C12 myoblasts and human HeLa_705 cells.

## Discussion

Phosphorothioate modification of 2OMe is promising therapeutically and can create more effective drugs for antisense oligonucleotide-targeted treatments. The optimisation of PS inclusion in 2OMe could improve its splicing efficiency in several different tissue types, and aid a deeper understanding of the pharmacological effects and side effects of 2OMePS (Flanigan et al., 2014).

Three-terminal PS modifications (2OMe-endPS) are sufficient to give full exonuclease stability to 2OMe despite showing reduced uptake, cellular distribution, and negligible nuclear localization, and splice-switching activity in several muscle cell lines and reporter cells. On the other hand, splice switching was highly enhanced by the full PS modification in 2OMe, in a relatively dose-dependent manner, with subtle differences pertaining to SSO length and modification between different cell types. Using lipofection, 2OMePS was rapidly taken up by the cells that are primarily accumulated in the nucleus. Following gymnotic uptake, 2OMePS displayed a predominant cytosolic distribution, and a relatively shorter length (20 mers) of SSO was vital in facilitating the uptake and activity in mouse myoblasts because 25 mers had almost zero activity. Accordingly, we confirmed that full PS modification could facilitate the uptake of 2OMe in different cellular splice-switching models of neuromuscular, hepatic, and bone diseases. Critically, it has been shown that the cellular distribution of 2OMePS could be regulated in the nucleus upon lipofection and the cytosol after gymnotic uptake, highlighting that the mechanism of cell entry is crucial for correct delivery-efficient splice switching.

In this study, we have confirmed Ncl protein to be a key player in this regulation, substantiating previous findings. Ncl has multiple proposed functions within and outside of the nucleoli. For example, it is involved in ribosome biogenesis, including the first processing step of pre-ribosomal RNA (rRNA) maturation, DNA repair, mRNA metabolism, an internalization of growth significant factors and viral ligands, and virus replication (Orrick et al., 1973). It has also been reported that Ncl and nucleophosmin disperse into the nucleoplasm during heat shock stress and reaccumulate in the nucleolus within an hour after release from the stress in normal cells; however, this reaccumulation of Ncl is strongly inhibited in cells lacking Hikeshi, a nuclear import carrier for Hsp70s (Kose et al., 2012).

It has been shown that 2OMePS enters the cells *via* endocytosis and accumulates in endosome-related structures in the cytoplasm (Marcusson et al., 1998). The interaction between Ncl and 2OMePS affected the potency of 2OMePS. The depletion of Ncl enhanced the splice-switching activity of 2OMePS, and our dissociation data suggest that the protein may decrease the splice-switching activity through direct interaction with 2OMePS (Figures 4C,E). These findings support the hypothesis that Ncl functions in the form of a heteromultimeric receptor complex (signalosomes) that may include other chaperones such as Tcp1 and other receptor proteins, including scavenger receptors (Ezzat et al., 2015). Tcp1 complex proteins are reported to interact with 2OMePS and colocalise in oligonucleotide-induced nuclear bodies in mammalian cells (Liang et al., 2014), but we were unable to reproduce these data in this study. It should be noted that Ncl shuttles between the nucleus, cytoplasm, and cell surface, and has been implicated in controlling regulatory processes and may play a role in pathogen infection and autoimmune diseases (Chen et al., 2008).

Furthermore, Ncl was reported to act as a scavenger receptor for acetylated low-density lipoproteins on macrophages (Canton et al., 2013; Ezzat et al., 2015; Miki et al., 2015), and other receptor proteins may be involved in signalosomes. Thus, it is possible that Ncl interacts with 2OMePS, leading to reduced 2OMePS activity upon diminishing of a particular 2OMe-binding protein. How Ncl reduces 2OMePS activity is still unclear. The binding of Ncl to 2OMePS likely prevents the binding of other proteins that would enhance its SSO activity. Ncl may be involved in the nuclear import of 2OMePS or simply act as a sequestering factor for 2OMePS competing with binding to its target RNA. In addition, the protein may also play a role in 2OMePS uptake and/or in the exchange between endocytosis-related organelles and the cytosol environment. Finally, we cannot rule out the possibility that Ncl may help to melt the intramolecular structures of 2OMePS to increase their base-pairing potential with target RNAs (Tosoni et al., 2015). Similarly, a minor modification to the 2OMePS may help circumvent this barrier. Proteomics following PAR-CLIP was only performed using one type of 2OMePS in C2C12 wild-type cells, not a disease model. These results do not show that DMD disease models would uptake SSOs and process them in the same way. Thus, the optimisation of the chemistry is crucial for the targeted tissue delivery, uptake, cellular localization, and effective splice-switching activity.

Ncl depletion could enhance the activity of 2OMePS and facilitate the cellular uptake of 2OMePS from extracellular environments and/or the release of 2OMePS from endosomes or lysosomes. Further investigation of the underlying mechanisms of how Ncl depletion improves 2OMePS drug potency will be necessary for a better drug design. It would be interesting to test if NCL aptamer, AS1411 (Carvalho et al., 2019), or surface NCL antagonists, HB-19-N6L (Verhaart and Aartsma, 2019), may affect 2OMePS and other SSOs' function.

## Conclusion

We found that PS modification facilitates the gymnotic uptake of 2OMe in myoblasts. We also found that splice switching is dependent on PS inclusion in 2OMe upon the lipofection in muscle, neuronal, liver, and bone cell lineages. Ncl could interact with 2OMePS and influence cellular uptake and intracellular distribution and display differential activity depending on the RNA target.

## Data Availability Statement

The raw data supporting the conclusions of this article will be made available by the authors, without undue reservation.

## Author Contributions

SA, YA, MB, and MW: conceptualization. YA, HJ, IM, MB, and TR: methodology. MA, CSJR, CS, TL, YH, JN, SM, and IM: investigation. YA: writing the original draft. TR, TL, CS, JN, MG, and SA: writing the review and editing. SA, YA, MW, and TL: funding acquisition. SA and MW: supervision. All authors contributed to the article and approved the submitted version.

## Funding

SA was supported by the Swedish Research Council and the Swedish Society of Medical Research (SSMF). This work was supported by the Swedish Medical Research Council, the MRC Confidence in Concept award (grant to YA and MW), the Japan Society for the Promotion of Science Grant-in-Aid for Scientific Research (C) (Grant No. 18K07544 to YA), Grants-in-Aid for Research on Nervous and Mental Disorders (Grant No. 2–6 to YA), the Japan Agency for Medical Research and Development (AMED) (Grant Nos. 19ek0109239h0003 and 19lm0203069h0002 to YA), and the Estonian Research Council (Grant No. PSG226 to TL).

## Conflict of Interest

MA was employed by Integrated DNA Technologies, Inc. The remaining authors declare that the research was conducted in the absence of any commercial or financial relationships that could be construed as a potential conflict of interest.

## Publisher's Note

All claims expressed in this article are solely those of the authors and do not necessarily represent those of their affiliated organizations, or those of the publisher, the editors and the reviewers. Any product that may be evaluated in this article, or claim that may be made by its manufacturer, is not guaranteed or endorsed by the publisher.
