# Supplementary material for: Fine Tuning of Phosphorothioate Inclusion in 2′-O-Methyl Oligonucleotides Contributes to Specific Cell Targeting for Splice-Switching Modulation
Source: Front Physiol. 2021 Oct 13;12:689179. doi: 10.3389/fphys.2021.689179 (PMC8548633; doi:10.3389/fphys.2021.689179)
Supplement: Supplementary file 1 [file Data_Sheet_1.pdf]

Supporting Information

Fine-tuning of phosphorothioate inclusion in 2'-O-methyl oligonucleotides contributes to specific cell targeting for splice-switching modulation

Yoshitsugu Aoki[1,2]*, Cristina S.J. Rocha[3], Taavi Lehto[3], Shouta Miyatake[1], Henrik Johansson[4], Yasumasa Hashimoto[1], Joel Z. Nordin[1,3], Imre Mager[2], Misako Aoki[2], McClorey Graham[2], Chaitra Sathyaprakash[1], Thomas C. Roberts[2], Matthew J.A. Wood[2], Mark A. Behlke[5], and Samir El Andaloussi[2,3],*

1 Department of Molecular Therapy, National Institute of Neuroscience, National Center of Neurology and Psychiatry (NCNP), Tokyo, Japan
2 Department of Paediatrics, University of Oxford, South Parks Road, Oxford, United Kingdom
3 Department of Laboratory Medicine, Clinical Research Center, Karolinska Institutet, Karolinska University Hospital, Huddinge, Sweden
4 Department of Oncology-Pathology, Clinical Research Center, Karolinska Institutet, Karolinska University Hospital, Huddinge, Sweden
5 Integrated DNA Technologies, Inc., Coralville, Iowa

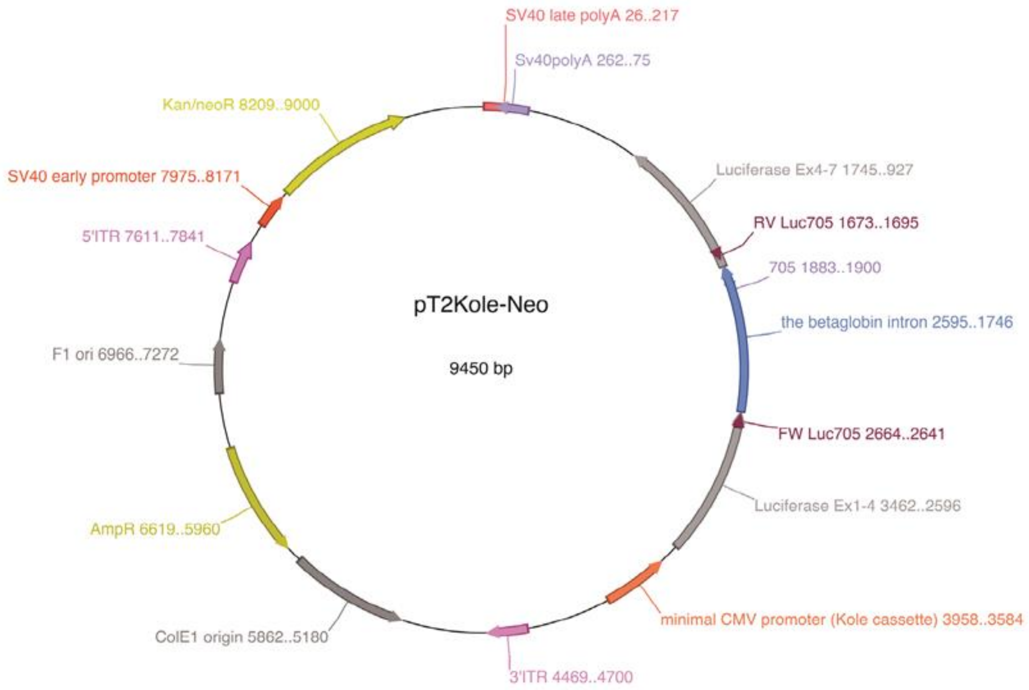

Figure S1. Schematic representation of the full reporter construct pT2Kole-Neo.

(Rocha et al. Nucleic Acid Ther. 2016;26:381-391.)

A

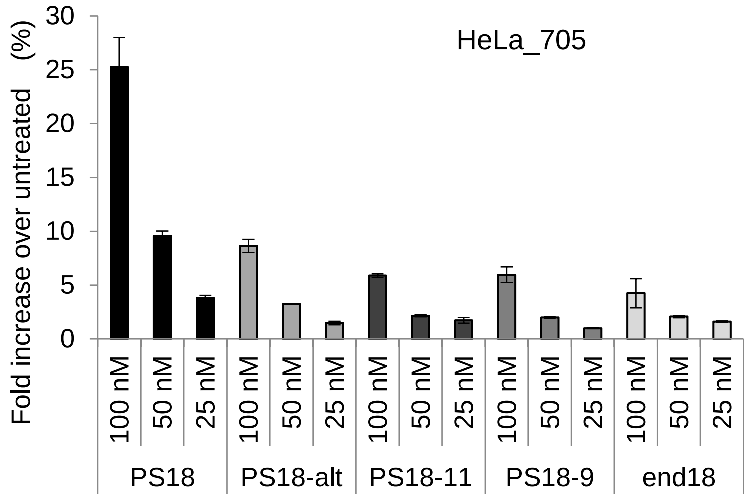

B

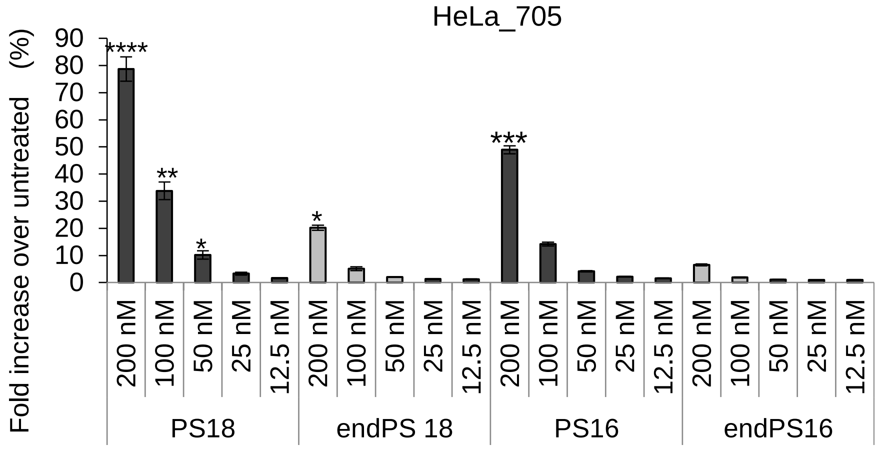

Figure S2. Phosphorothioate inclusion in 2'-O-methyl oligonucleotides on splice switching in HeLa_705 cells. **(A)** Luciferase production following splice correction with 18-mer modified with full PS (PS18) vs endPS (endPS18) 2OMe in the cells when transfected with different numbers of PS modifications at concentrations of 25, 50 or 100 nM. **(B)** Luciferase production following splice correction in the cells when transfected with PS18, endPS18, PS16 or endPS16 2OMe at concentrations of 12.5, 25, 50, 100 or 200 nM. P < 0.05 was defined as statistically significant. * P < 0.05; ** P ≤ 0.01, *** P ≤ 0.001; **** P ≤ 0.0001.

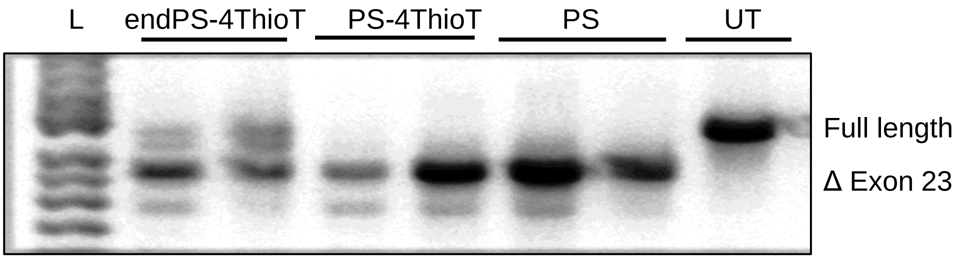

Figure S3. Photoactivatable 2OMe-endPS and 2OMePS modified with biotin (200 nM as final concentration) induced exon 23-skipping in C2C12 myoblasts. endPS-4ThioT: 5' biotin-2OMe-endPS-4ThioT-3’, PS-4ThioT: 5' biotin-2OMePS-4ThioT-3’, PS: 2OMePS, L: DNA ladder, UT: Untreated.

A

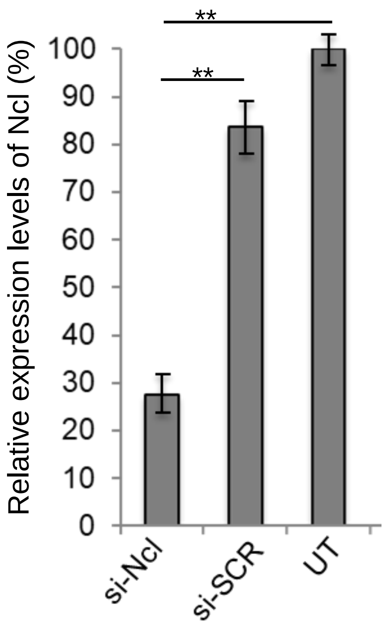

B

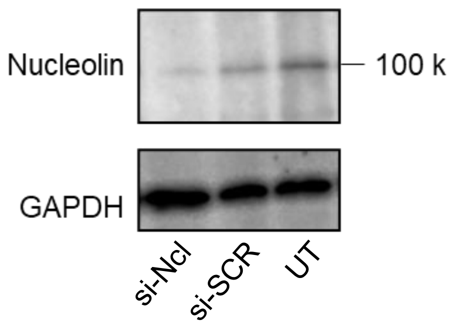

Figure S4. Nucleolin (Ncl) knockdown by siRNA in C2C12 myoblasts. The cells are incubated with siRNA for 24 hours in C2C12 myoblasts. A) Ncl knockdown levels by siRNA are evaluated using qPCR. Si-Ncl: Ncl siRNA treated cells, si-SCR: scramble siRNA treated cells, UT: untreated cells. B) Representative western blotting picture of Ncl in C2C12 myoblasts. Rabbit polyclonal anti-Nucleolin antibody (ab22758) is used for the study. $P < 0.05$ was defined as statistically significant. ** $P \leq 0.01$, error bars represent mean $\pm$ SEM.
